# Integrating implementation science to explore barriers and facilitators influencing nurses’ bioterrorism preparedness: a mixed-methods systematic review protocol

**DOI:** 10.3389/fpubh.2026.1887736

**Published:** 2026-06-29

**Authors:** Yiwei Luo, Qun Xiao, Bowen Li, Yanyan Zhang, Yanhui Liu, Chen Zhi, Qiushi Zhang, Minye Li, Hui Ma

**Affiliations:** 1 Department of Nursing, Chinese PLA General Hospital, Beijing, China; 2 School of Nursing, Southern Medical University, Guangzhou, China; 3 Respiratory and Critical Care Medicine, The First Medical of Chinese PLA General Hospital, Beijing, China; 4 School of Nursing, Tianjin University of Traditional Chinese Medicine, Tianjin, China; 5 Joint Logistics Academy, National Defense University of PLA, Beijing, China

**Keywords:** barriers and facilitators, bioterrorism preparedness, implementation science, mixed-methods systematic review, nurses

## Abstract

**Background:**

Bioterrorism involves the intentional release of biological agents capable of causing mass casualties, public health emergencies, and societal disruption. As frontline healthcare providers, nurses play a critical role in early detection, isolation, emergency care, and surveillance. However, substantial gaps exist in nursing bioterrorism preparedness across healthcare settings, and implementation determinants influencing preparedness remain insufficiently synthesised.

**Objective:**

To systematically synthesise quantitative and qualitative evidence on barriers and facilitators influencing nurses’ bioterrorism preparedness using the Consolidated Framework for Implementation Research (CFIR 2.0).

**Methods:**

This mixed-methods systematic review will follow the Joanna Briggs Institute (JBI) methodology for mixed-methods systematic reviews and adhere to PRISMA-P guidelines, with CFIR 2.0 serving as the guiding analytical framework. A comprehensive literature search will be performed across CINAHL, PubMed, Embase, Scopus, Web of Science, ProQuest, Cochrane Library, and Medline electronic databases from their inception until March 2026, including English and Chinese publications. Two independent reviewers will screen identified records against predefined eligibility criteria and appraise the methodological quality of included studies using the Mixed Methods Appraisal Tool (MMAT). Data synthesis will be thematically mapped to CFIR 2.0 constructs and integrated using a convergent integrated approach.

**Anticipated contributions:**

This review is expected to provide the first synthesis informed by implementation science of determinants influencing nurses’ bioterrorism preparedness. By integrating evidence across methodological traditions, the review will generate a comprehensive understanding of individual, organisational, and contextual factors shaping preparedness implementation.

**Discussion:**

This protocol describes a mixed-methods systematic review informed by implementation science and guided by the Consolidated Framework for Implementation Research (CFIR 2.0) to examine barriers and facilitators influencing nurses’ bioterrorism preparedness. The findings are expected to support a more comprehensive understanding of factors shaping preparedness in healthcare settings and to inform evidence-informed preparedness planning, nursing education, and organisational support strategies aimed at strengthening healthcare system resilience to biological threats.

**Systematic review registration:**

https://www.crd.york.ac.uk/PROSPERO/view/CRD420251273403, Identifier CRD420251273403.

## Introduction

Bioterrorism, defined as the deliberate release of biological agents to cause death or disease in humans, animals or plants, represents a distinct and serious threat to public health and healthcare system resilience (1). Unlike naturally occurring outbreaks, biological attacks are characterised by high concealment, the potential for silent propagation over days or weeks before detection, and the capacity to severely compromise healthcare systems before the threat is identified (2, 3). The growing international attention to bioterrorism preparedness reflects both the rising incidence of global infectious diseases and the increasingly volatile nature of international security (4, 5).

As the largest occupational group within the healthcare system, nurses serve as the first responders during bioterrorism incidents. Their role is critical in early symptom recognition, the implementation of isolation measures, the provision of emergency care, public health surveillance, and psychological support (6, 7). However, significant gaps remain in nurses’ bioterrorism preparedness. Key response competencies, such as the proper use of Personal Protective Equipment (PPE), targeted infection control, and the safe handling of high-risk pathogens, are frequently reported as inadequate in existing training curricula (8–11). Beyond technical skills, frontline nurses encounter profound challenges, including ethical dilemmas, insufficient specialised training for identifying rare agents, and conflicting priorities between routine clinical care and emergency response. Psychologically, they face the fear of transmitting infections to their families, moral distress, and burnout stemming from intense workloads, all of which compromise their well-being and decision-making capabilities (12). Evidence suggests that nurses’ willingness to respond and their preparedness levels directly correlate with the overall effectiveness of emergency outcomes (13). Therefore, enhancing nurses’ bioterrorism preparedness is fundamental to maintaining the resilience and stability of the public health system (10, 14).

Despite increasing research attention, existing studies on nurses’ bioterrorism preparedness remain largely fragmented, existing literature consistently indicates that nurses are generally inadequately prepared for bioterrorism events (9, 15). While quantitative research can elucidate the prevalence of preparedness levels and associated influencing factors, qualitative studies provide deeper insights into nurses’ perceptions, motivations, and experiences (10). For instance, Jeno (10) identified perceived barriers and facilitators among emergency nurses through qualitative analysis, while Thoms (16) validated the effectiveness of self-learning modules for improving preparedness through quantitative investigation. Combining quantitative and qualitative evidence may therefore provide a broader and more actionable understanding of the factors influencing nurses’ bioterrorism preparedness.

Despite increasing attention to disaster and emergency preparedness, existing studies on nurses’ bioterrorism preparedness predominantly focus on preparedness levels, knowledge, attitudes, or willingness to respond, without systematically examining the implementation determinants that shape preparedness across healthcare settings. Preparedness is shaped not only by individual competencies but also by organisational culture, leadership support, training infrastructure, resource availability, and policy environments. An implementation science perspective is therefore needed to understand how preparedness interventions are adopted, implemented, and sustained in practice.

This protocol describes a mixed-methods systematic review underpinned by CFIR 2.0 (17), which organises implementation determinants across five domains: intervention characteristics, outer setting, inner setting, characteristics of individuals, and process. By integrating quantitative and qualitative evidence within this framework, this study seeks to provide an actionable synthesis of multilevel implementation determinants to inform future policy-making and educational strategies.

## Objective

The primary aim of this mixed-methods systematic review is to synthesise implementation determinants influencing nurses’ bioterrorism preparedness across healthcare contexts using the CFIR 2.0 as an analytical framework. Specifically, the review aims: (1) to systematically synthesise evidence from quantitative, qualitative, and mixed methods studies to identify the barriers and facilitators influencing the implementation of nurses’ bioterrorism preparedness and (2) to categorise these identified barriers and facilitators according to the five domains of the CFIR 2.0, thereby providing evidence-based recommendations for the development of targeted administrative policies, educational interventions, and clinical support strategies.

## Method

### Protocol and registration

This protocol describes a mixed-methods systematic review following the JBI methodology for mixed-methods systematic reviews using a convergent integrated approach (18) and Preferred Reporting Items for Systematic Reviews and Meta-Analyses Protocols (PRISMA-P) 2015 statement (19) (Appendix C). It is registered with PROSPERO (registration ID: CRD420251273403; Available from: https://www.crd.york.ac.uk/PROSPERO/view/CRD420251273403.) This protocol is not an amendment of a previously completed or published protocol. Any important amendments made during the review process will be documented and reported in the final review publication and updated in the PROSPERO registration record where appropriate.

### Search strategy

The strategy will follow JBI three-step search approach for mixed-methods systematic reviews. First, an initial limited search of PubMed and CINAHL will be undertaken to identify relevant articles and examine text words contained in titles and abstracts, as well as index terms and Medical Subject Headings (MeSH). Second, identified keywords and controlled vocabulary terms will be adapted and applied across all included electronic databases. Third, the reference lists of all included studies and relevant review articles will be manually screened to identify additional eligible studies.

The search strategy will combine controlled vocabulary terms (MeSH and Emtree terms) and free-text keywords related to four core concepts: (1) nurses, (2) bioterrorism or biological emergencies, (3) preparedness, emergency response, and (4) implementation determinants. To improve search sensitivity, implementation science-related terms such as “barriers,” “facilitators,” “implementation,” “leadership support,” “organisational readiness,” and” emergency response” will also be incorporated where appropriate.

An integrated literature search will be conducted in the following electronic databases from inception to March 2026: CINAHL, PubMed, Embase, Scopus, Web of Science, ProQuest, Cochrane Library, and MEDLINE. Grey literature will additionally be searched through ProQuest Dissertations and Theses Global, Open Access Theses and Dissertations, World Health Organization (WHO) reports, national Centers for Disease Control and Prevention (CDC) documents, and reports from international nursing and public health organisations.

The search will include studies published in English and Chinese. Articles published in other languages that appear potentially eligible during title and abstract screening will be assessed using DeepL Translator; full-text translation will only be pursued where the study appears highly relevant and the machine translation output is judged sufficiently coherent for accurate interpretation by a bilingual reviewer. Translated findings will be clearly flagged during data synthesis. Search results from all databases will be exported into EndNote 20 for reference management and duplicate removal prior to screening. The complete search strategies for all databases will be provided in Appendix A.

### Eligibility criteria

#### Inclusion criteria

Type of studies: Quantitative research (cross-sectional surveys, cohort studies, case–control studies), qualitative research (phenomenological research, grounded theory, case studies, focus group interviews), and mixed-methods studies.

Participants: Registered nurses, nursing students, and nurse managers.

Phenomena of interest: This review will focus on implementation determinants shaping nurses’ bioterrorism preparedness, including barriers and facilitators associated with preparedness efforts, training, response capacity, and organisational readiness.

Outcomes: Primary outcomes focused on nurses’ bioterrorism preparedness, including knowledge, attitudes, skills, willingness to respond, self-efficacy, and training experiences. Studies explicitly reporting barriers to or facilitators of preparedness are prioritised. Secondary outcomes include organisational policies, resource availability, and system-level factors influencing preparedness implementation.

Context: medical or public health settings (hospitals, emergency centers, community health services, nursing schools).

#### Exclusion criteria

Exclusion criteria are: (1) Studies focusing on non-nurse populations (e.g., physicians, medical students) or where nurse-specific data cannot be disaggregated. (2) Editorials, letters, conference abstracts, and non-systematic reviews.

#### Selection process

Retrieved records will be imported into EndNote 20 for deduplication. Two reviewers (XQ and CJQ) will independently screen titles and abstracts, followed by full-text assessment against the eligibility criteria using a standardised form. Discrepancies will be resolved through consensus; unresolved conflicts will be adjudicated by a third reviewer (LYW). The selection process will be documented in a PRISMA 2020 flow diagram, including reasons for full-text exclusions. In cases of confirmed overlap, when multiple publications present findings originating from the same or overlapping study populations, a comparison will be conducted regarding publication characteristics, study settings, sample descriptions, recruitment periods, and author information. Where overlap is confirmed, the most comprehensive report will be retained, while supplementary reports will be used only when they provide unique data relevant to the review objectives.

### Data extraction

A standardized data extraction form will be developed and pilot-tested prior to formal data extraction. The form is informed by CFIR 2.0 and the Joanna Briggs Institute methodology for mixed-methods systematic reviews. The extraction framework is designed to systematically capture study characteristics, preparedness outcomes, and implementation determinants related to nurses’ bioterrorism preparedness (Appendix B).

The primary outcomes of interest are barriers and facilitators influencing nurses’ bioterrorism preparedness. These factors will be extracted from quantitative, qualitative, and mixed-methods studies and subsequently mapped to the domains and constructs of the Consolidated Framework for Implementation Research (CFIR 2.0). Additional outcomes will include preparedness-related measures reported by the included studies, such as knowledge, attitudes, self-efficacy, willingness to respond, competency, perceived preparedness, and organisational readiness, where these outcomes contribute to understanding implementation determinants. Study characteristics, participant characteristics, healthcare setting, and contextual information relevant to preparedness implementation will also be extracted.

Two reviewers (XQ and CJQ) will extract data independently; disagreements will be resolved via discussion or third-party adjudication (LYW). Any discrepancies will be documented and resolved through discussion. Where consensus cannot be reached, a third reviewer will be consulted to adjudicate the decision. The number and nature of disagreements identified during the review process will be recorded to ensure transparency and auditability of study selection and data extraction decisions.

### Quality assessment

Methodological quality will be appraised using the Mixed Methods Appraisal Tool (MMAT) 2018 (20). Two researchers (LYW and XQ) will independently evaluate the included studies. The tool assesses the rigour of study design, the appropriateness of sampling methods, the validity of data collection and analysis, and completeness of reporting. Each study will be assessed against design-specific criteria, with ratings of “Yes,” “No” or “Cannot tell.” Consistent with MMAT guidance, studies will not be excluded based on an overall numerical score. Instead, methodological limitations will be considered during data synthesis and interpretation of findings. Methodological limitations of included studies will be narratively synthesised to discuss their potential impact on the findings.

### Synthesis methods

Data synthesis will follow the convergent integrated approach according to JBI methodology. This approach was selected to enable the systematic integration of quantitative, qualitative, and mixed-methods evidence into a unified implementation science-inform synthesis of determinants influencing nurses’ bioterrorism preparedness. As studies are anticipated to employ diverse methodologies and outcome measures, quantitative findings will first undergo qualitization, whereby numerical results and statistical associations are transformed into narrative statements reflecting their substantive meanings, guided by the framework described (21). Two reviewers will independently conduct qualitization, and discrepancies in narrative rendering will be resolved through discussion. Percentage agreement will be calculated and reported at each stage of screening, quality appraisal, and data extraction as a descriptive indicator of consistency. Disagreements will be resolved through structured discussion, and if consensus cannot be reached, a third reviewer will be consulted. This process allows quantitative evidence to be integrated directly with qualitative findings while preserving the contextual interpretation of preparedness determinants. No hierarchical weighting will be assigned to qualitative or quantitative evidence during integration. Following data transformation, extracted findings will undergo a combined deductive–inductive thematic synthesis guided by the CFIR 2.0.

Initially, identified barriers and facilitators will be deductively mapped onto the five domains and relevant constructs. This deductive stage will provide a structured framework for organising implementation determinants across studies and healthcare contexts. Subsequently, inductive coding will be conducted within and across CFIR domains to identify emergent themes, contextual nuances, and unanticipated determinants not fully captured by predefined constructs. This iterative process will allow both theory-informed interpretation and data-driven theme development, allowing additional context-specific themes to emerge.

Integrated findings will then be synthesised narratively to develop an integrated understanding of the multilevel determinants influencing nurses’ bioterrorism preparedness across healthcare settings. Evidence will be triangulated across study designs, settings, and participant groups to identify patterns of convergence, divergence, complementarity, and contextual variation. However, given the anticipated heterogeneity in study designs, outcome measures, and implementation contexts, meta-analysis is not planned. The final synthesis will generate an evidence-informed conceptual understanding of how multilevel implementation determinants interact to shape nurses’ bioterrorism preparedness across healthcare systems and emergency preparedness contexts.

To ensure coding consistency and methodological rigour, two reviewers (LYW and XQ) will independently perform coding and CFIR 2.0 mapping. Prior to full synthesis, reviewer calibration exercises will be conducted using a subset of included studies to refine coding consistency and operational interpretation of CFIR constructs. Disagreements in coding or construct allocation will be resolved through discussion, with consultation from a third reviewer (CJQ) when necessary.

## Anticipated contributions

**Figure 1:**
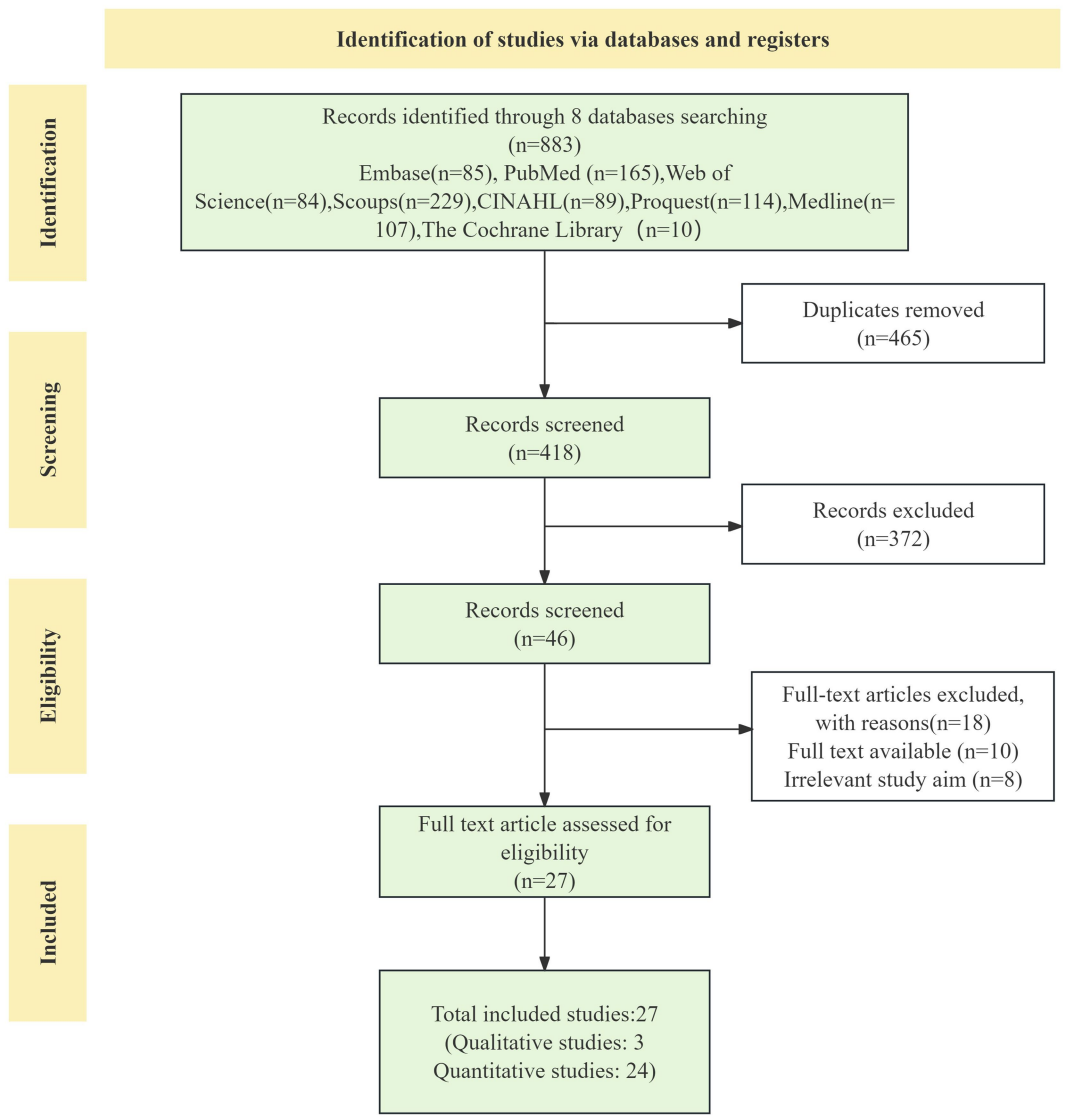
PRISMA 2020 flow diagram of study selection.

This review is expected to bring together fragmented evidence on nurses’ bioterrorism preparedness from both quantitative and qualitative research. By applying the CFIR2.0 framework, this review will examine how factors such as leadership support, workplace culture, resource availability, communication systems, and training environments shape preparedness across healthcare settings. Integrating evidence from different methodological traditions may also help clarify why preparedness interventions are difficult to implement or sustain in some contexts despite increasing awareness of biological threats. The findings may be useful for nursing educators, hospital administrators, and policymakers seeking to strengthen preparedness planning and workforce support. This review may also contribute to the growing use of implementation science approaches in disaster nursing research by demonstrating how CFIR can be applied to understand preparedness beyond individual-level capability alone. The literature search has been completed, and the specific process and search results are shown in Figure 1.

## Discussion

This review is expected to address a substantive gap in the existing literature by synthesising evidence on barriers and facilitators influencing nurses’ bioterrorism preparedness across healthcare settings. Although preparedness has been widely discussed in disaster and emergency nursing (2, 22), less attention has been paid to the implementation conditions that influence preparedness in practice (7, 8, 23). This review therefore seeks to apply CFIR 2.0 to move beyond competency-focused perspectives and provide a structured, multilevel understanding of preparedness implementation and sustainability.

By applying the CFIR 2.0, this review is expected to produce findings that extend beyond a descriptive account of preparedness levels. The five domains of CFIR 2.0 provide a structured lens through which barriers and facilitators can be systematically identified and contextualised across studies and healthcare settings (24). This strategy is anticipated to reveal how factors such as institutional leadership, organisational readiness, communication systems, and training infrastructure interact to enable or constrain preparedness implementation, offering a level of analytical depth that individual studies have not yet achieved (25–27).

The mixed-methods design may also help capture dimensions of preparedness that are difficult to understand through quantitative findings alone. For example, qualitative evidence may provide insight into how nurses experience ethical tension, uncertainty, fear of infection transmission to family members, or emotional exhaustion during biological emergencies. At the same time, quantitative evidence may help identify recurring patterns related to training exposure, preparedness confidence, or willingness to respond. Bringing these forms of evidence together may provide a more practice-oriented understanding of preparedness implementation.

The findings of this review may have implications for preparedness education and organisational planning. Simulation-based training, interdisciplinary exercises, and scenario-based learning have been increasingly recommended in emergency preparedness education (28, 29). However, training effectiveness may depend not only on educational content but also on whether healthcare organisations provide sufficient time, staffing support, leadership engagement, and opportunities for ongoing practice. Similarly, preparedness policies may be difficult to operationalise in environments characterised by workforce shortages, competing clinical priorities, or limited institutional resources. The review may also provide insights into the potential role of digital technologies, such as virtual reality drills and mobile learning platforms, in creating immersive, safe environments for training on high-risk, low-frequency events (30)^.^ The findings of this review may inform recommendations for strengthening resource allocation, developing supportive leadership structures, and formulating clear clinical practice guidelines to help mitigate ethical dilemmas and workload pressures during crises (31, 32).

Rather than viewing preparedness solely as an individual competency issue, the review seeks to examine how preparedness is supported, constrained, and maintained within real-world healthcare environments. Several limitations should be acknowledged. First, although articles in other languages that appear potentially eligible will be assessed using DeepL Translator at the screening stage, with full-text translation pursued only where the study appears highly relevant and the translation output is judged sufficiently coherent by a bilingual reviewer, the reliability of machine translation cannot be fully assured, and translated findings will therefore be treated with appropriate caution and clearly flagged during synthesis. Second, the deductive application of CFIR 2.0 constructs carries an inherent risk of confirmation bias, whereby findings may be mapped onto predefined categories even when the data more accurately reflect emergent or context-specific phenomena. To mitigate this, inductive coding will be conducted in parallel with deductive mapping, allowing additional themes to surface beyond the predefined framework, and all coding discrepancies will be systematically documented and resolved through discussion. Third, the convergent integrated approach assumes that qualitative and quantitative evidence can be meaningfully integrated, although differences in epistemological assumptions and methodological traditions may influence interpretation. Fourth, despite efforts to search grey literature sources, unpublished evidence may remain underrepresented. Finally, variation in study designs, preparedness definitions, and outcome measures across included studies may limit direct comparability, and findings should therefore be interpreted with this heterogeneity in mind.

## Conclusion

This protocol describes a mixed-methods systematic review informed by implementation science and guided by CFIR 2.0 to examine barriers and facilitators influencing bioterrorism preparedness across healthcare settings. By integrating evidence from quantitative, qualitative, and mixed-methods studies, the review aims to produce a structured synthesis of implementation determinants operating at individual, organisational, and system levels. The anticipated outputs include a thematic mapping of barriers and facilitators onto CFIR 2.0 domains and evidence-informed recommendations for nursing education, organisational preparedness planning, and policy development.

The findings are intended to support a more contextually grounded understanding of why preparedness efforts succeed or falter in practice, moving beyond individual competency to account for the institutional and structural conditions in which nurses work. By identifying determinants relevant to the adoption, implementation, and sustainability of preparedness interventions, this review may provide useful evidence for healthcare administrators, nursing educators, and policymakers in developing more targeted and context-sensitive strategies. It may contribute modestly but meaningfully to strengthening workforce preparedness and broader healthcare system resilience in response to biological threats and other public health emergencies.

## Supplementary material

Supplementary File 1 Search strategy for all databases included in the review. Detailed search strategies, including search terms, Boolean operators, and database-specific adaptations.

Supplementary File 2 Standardized data extraction form for eligible studies. Template used to extract study characteristics, methodological information, and relevant data items from included studies.
