# Supplementary material for: Integrating implementation science to explore barriers and facilitators influencing nurses’ bioterrorism preparedness: a mixed-methods systematic review protocol
Source: Front Public Health. 2026 Jun 29;14:1887736. doi: 10.3389/fpubh.2026.1887736 (PMC13357410; doi:10.3389/fpubh.2026.1887736)
Supplement: Supplementary File 1 — Search strategy for all databases included in the review. Detailed search strategies, including search terms, Boolean operators, and database-specific adaptations. [file Supplementary_file_1.DOCX]

**Appendix A: Search Strategy**

**Example Search Strategy**

#1 Nurses

("Nurses"[Mesh] OR nurs*[tiab] OR "registered nurse*"[tiab]

OR "nursing staff"[tiab] OR "nursing personnel"[tiab])

#2 Bioterrorism and biological emergencies

("Bioterrorism"[Mesh] OR bioterrorism[tiab]

OR "bioterrorist attack*"[tiab]

OR "biological threat*"[tiab]

OR "biological emergenc*"[tiab]

OR "biologic* emergenc*"[tiab]

OR "biological weapon*"[tiab])

#3 Preparedness and emergency response

("Disaster Planning"[Mesh]

OR preparedness[tiab]

OR readiness[tiab]

OR "disaster preparedness"[tiab]

OR "emergency preparedness"[tiab]

OR "emergency response"[tiab]

OR "response capacity"[tiab])

#4 Implementation determinants

(barrier*[tiab]

OR facilitator*[tiab]

OR implementation[tiab]

OR "implementation science"[tiab]

OR "organizational readiness"[tiab]

OR "organisational readiness"[tiab]

OR "leadership support"[tiab]

OR "workplace culture"[tiab]

OR "contextual factor*"[tiab]

OR "implementation determinant*"[tiab])

#5 Final search

#1 AND #2 AND #3 AND #4

*The final database search will be conducted prior to the completion of the review to identify newly published studies.*
