# Supplementary material for: Integrating implementation science to explore barriers and facilitators influencing nurses’ bioterrorism preparedness: a mixed-methods systematic review protocol
Source: Front Public Health. 2026 Jun 29;14:1887736. doi: 10.3389/fpubh.2026.1887736 (PMC13357410; doi:10.3389/fpubh.2026.1887736)
Supplement: Supplementary File 2 — Standardized data extraction form for eligible studies. Template used to extract study characteristics, methodological information, and relevant data items from included studies. [file Supplementary_file_2.DOCX]

**Appendix B: Data Extraction**

**Table 1** General characteristics

| **Item** | **Description** |
| --- | --- |
| Study ID | Author(s), year |
| Country/Region | Country or geographical setting |
| Study aim | Research objective(s) |
| Study design | Quantitative, qualitative, or mixed-methods |
| Study setting | Hospital, emergency department, community health centre, nursing school, military setting, etc. |
| Participant characteristics | Type of nurses, sample size, years of experience, specialty |
| Context | Clinical/public health/emergency preparedness context |
| Data collection method | Survey, interview, focus group, observation, etc. |
| Theoretical framework used | Any reported theoretical/model framework |
| Key preparedness domain | Knowledge, skills, willingness, self-efficacy, training, response capacity |

**Table 2** Preparedness-related outcomes

| **Outcome category** | **Extracted data** |
| --- | --- |
| Knowledge preparedness | Findings related to knowledge of bioterrorism agents, protocols, infection control |
| Clinical preparedness | Isolation, PPE use, triage, emergency response competency |
| Psychological preparedness | Fear, anxiety, moral distress, burnout |
| Willingness to respond | Intention or willingness to participate in response |
| Training experiences | Participation in drills, simulations, educational interventions |
| Organisational preparedness | Institutional support, emergency planning, communication systems |
| System-level preparedness | Resource availability, interprofessional coordination, policy support |

**Table 3** CFIR-based implementation determinants extraction

| **CFIR Domain** | **CFIR construct** | **Operational definition** | **Barriers** | **Facilitators** |
| --- | --- | --- | --- | --- |
| Innovation characteristics | Relative advantage | Perceived benefit of preparedness interventions compared with existing training or emergency procedures |  |  |
|  | Adaptability | Degree to which preparedness interventions can be adapted to different healthcare settings |  |  |
|  | Complexity | Perceived difficulty associated with preparedness protocols or interventions |  |  |
|  | Design quality and packaging | Quality and accessibility of preparedness education materials and interventions |  |  |
|  | Cost | Financial, workload, or time burden associated with preparedness implementation |  |  |
| Outer setting | \| nurses needs \|  \|  \| \| --- \| --- \| --- \| | Extent to which preparedness reflects community and public health needs |  |  |
|  | \| External policy and incentives \|  \|  \| \| --- \| --- \| --- \| | Influence of governmental policies, accreditation standards, and emergency regulations |  |  |
|  | \| Interorganizational networks \|  \|  \| \| --- \| --- \| --- \| | Degree of collaboration between organisations and emergency systems |  |  |
|  | \| Sociopolitical context \| \| --- \| | Broader social, political, and security environment influencing preparedness |  |  |
| Inner setting | \| Organisational culture \|  \|  \| \| --- \| --- \| --- \| | Institutional values and norms regarding emergency preparedness |  |  |
|  | \| Leadership engagement \|  \|  \| \| --- \| --- \| --- \| | Commitment and involvement of organisational leadership in preparedness implementation |  |  |
|  | \| Available resources \|  \|  \| \| --- \| --- \| --- \| | Availability of staffing, PPE, infrastructure, and educational resources |  |  |
|  | \| Learning climate \|  \|  \| \| --- \| --- \| --- \| | Organisational support for continuous learning and preparedness improvement |  |  |
|  | \| Readiness for implementation \| \| --- \| | Institutional preparedness and willingness to implement preparedness interventions |  |  |
|  | Communication climate | Effectiveness of internal communication during preparedness activities |  |  |
| Characteristics of individuals | \| Knowledge and beliefs \|  \|  \| \| --- \| --- \| --- \| | Nurses’ perceptions, understanding, and attitudes toward preparedness |  |  |
|  | \| Self-efficacy \|  \|  \| \| --- \| --- \| --- \| | Confidence in ability to perform preparedness-related tasks |  |  |
|  | \| Individual motivation \|  \|  \| \| --- \| --- \| --- \| | Personal willingness and professional commitment to respond |  |  |
|  | \| Professional identity \|  \|  \| \| --- \| --- \| --- \| | Perceived role responsibility during bioterrorism events |  |  |
|  | \| Stress and emotional burden \| \| --- \| | Psychological stress associated with emergency response |  |  |
| Implementation process | \| Planning \|  \|  \| \| --- \| --- \| --- \| | Degree of strategic preparedness planning before implementation |  |  |
|  | \| Engaging \|  \|  \| \| --- \| --- \| --- \| | Involvement of stakeholders in preparedness implementation |  |  |
|  | \| Executing \|  \|  \| \| --- \| --- \| --- \| | Carrying out preparedness interventions effectively |  |  |
|  | \| Reflecting and evaluating \| \| --- \| | Monitoring and evaluating preparedness activities and outcomes |  |  |

**Table 4** Mixed-methods integration

| **Item** | **Description** |
| --- | --- |
| Type of evidence | Quantitative / qualitative / mixed-methods |
| Main findings | Summary of preparedness-related findings |
| Qualitized quantitative findings | Narrative transformation of quantitative evidence |
| Integrated themes | Combined themes generated during synthesis |
| CFIR mapping summary | Final assigned CFIR domain/construct |
| Reviewer notes | Additional interpretation or contextual comments |
